# Development of an mHealth App Prototype for LGBTQIA+ Individuals’ Sexual and Reproductive Health in Gauteng Province, South Africa: Design Science Research Study

**DOI:** 10.2196/79593

**Published:** 2025-12-23

**Authors:** Raikane James Seretlo, Mathildah Mpata Mokgatle, Hanlie Smuts, Nombulelo Veronica Sepeng

**Affiliations:** 1 Department of Public Health, School of Health Care Sciences, Sefako Makgatho Health Sciences University, Molotlegi Street, Ga-Rankuwa, Pretoria, 0204, South Africa, 27 12 521 411; 2 Department of Informatics, Faculty of Engineering, Built Environment and Information Technology, University of Pretoria, Pretoria, South Africa; 3 Department of Nursing Science, Faculty of Health Sciences, University of Pretoria, Pretoria, South Africa

**Keywords:** mHealth app, prototype, sexual and reproductive health, LGBTQIA+, wellness, mobile health, Lesbian, Gay, Bisexual, Transgender, Queer, Intersex, Asexual+

## Abstract

**Background:**

The fast rate of technological advances in the health care sector remain as a pressing need for effective solutions that address the unique health care needs of sexual and gender minorities. If these innovative solutions are considered, societal challenges such as stigma, discrimination, and a lack of tailored health care resources, as experienced by the lesbian, gay, bisexual, transgender, queer, intersex, asexual, and more (LGBTQIA+) individuals could be addressed at lower cost.

**Objective:**

This study aimed to develop a mobile health (mHealth) app specifically designed to address the sexual and reproductive health (SRH) of lesbian, gay, bisexual, transgender, queer, intersex, asexual, and more (LGBTQIA+) individuals in Gauteng Province, South Africa.

**Methods:**

This study used a Design Science Research (DSR) framework and a mixed-method exploratory sequential approach. DSR was executed in three cycles: cycle one followed an ethnography approach; involved 33 health care providers (HCPs) and 22 LGBTQIA+ individuals, focusing on identifying specific SRH and the challenges encountered in accessing and providing these services. Participants shared their views on the potential role of mHealth apps in addressing these issues. Cycle two engaged 13 experts through Participatory Action Research (PAR) approach using the Nominal Group Technique (NGT) to collaboratively identify essential content for the app, fostering a co-creation process. Lastly, cycle three followed an interventional pre-experimental approach by involving software developers and principal investigator working together to develop a functional prototype of the mHealth app.

**Results:**

This study revealed critical insights into the specific SRH of LGBTQIA+ individuals, alongside the barriers faced by health care providers in meeting these needs. The co-created app prototype named *“Queery wellness hub”* was developed and incorporated features tailored to enhance accessibility, confidentiality, and user engagement, addressing both user and provider perspectives.

**Conclusions:**

The findings underscore the potential of mHealth apps in transforming the delivery of SRH for LGBTQIA+ individuals in South Africa. Continued collaboration with stakeholders is essential for further refinement and successful implementation of the app, ultimately contributing to better health outcomes for sexual and gender minorities.

## Introduction

Mobile health (mHealth) is the delivery of health care services through mobile device communications and technology such as mobile phones, monitoring of patients’ devices, and individual virtual assistants [1]. The adoption and increasing attention to mobile health technologies are rising across multiple sectors, including the health care sector [2,3]. This change penetrates and affects the world at large regardless of different countries’ economic status and is moving from the high-income countries (HICs) to the low-income countries (LICs) [1,4]. The aim of this study was to develop an mHealth app designed for health care professionals (HCPs) and LGBTQIA+ individuals in Gauteng Province, South Africa, to address SRH needs and improve the overall health and well-being of LGBTQIA+ individuals.

However, the question remains as to whether every country is ready for this trend of what is called “The digital era,” not only for the use and adoption of technological tools and solutions but also addressing the health care issues and burden of diseases for all. Nevertheless, several studies have shown that the use of technology and artificial intelligence (AI) can help to solve and address some of the health care problems. For example, Mirbabaie et al [5], demonstrated that AI is playing a significant role in enhancing the health care state, such as assisting with disease detection and diagnosis, personalized medicine [6], faster verification of the drug target, and optimizing drug structure design [7].

Research demonstrated that it improves health care availability and quality in a variety of global regions [1] and promotes efficiency in areas such as the teamwork process, making interprofessional communication more effective and accurate, lowering risks [8,9]. In addition, the use of mHealth influences clinical results, such as decreases in blood pressure, glycosylated hemoglobin (HbA_1c_), and cholesterol, and assists with improved compliance with medications and physical activity, and certain psychological difficulties, such as reduced anxiety and increased patient satisfaction, were found [9]. Lastly Rowland et al [10], showed that it aids with preventative behavior change, virtual self-management of a specific ailment, and diagnosis .

Furthermore, with regard to the health and wellbeing of LGBTQIA+ individuals, various studies have shown their use of various mHealth and innovative tools and solutions, there was a rise in pre-exposure prophylaxis (PrEP) adherence, a substantial rise in complete HIV knowledge and treatment, accessibility to sexual health knowledge about good sexual behaviors, and some were reminded about their HIV and sexually transmitted infections (STIs) tests [11-17].

SRH services remain one of the important needs of human beings for a holistic care approach. Access to sexual and reproductive health services is a fundamental human right that should be readily available to everyone at all times as part of a universal healthcare system [18]. However, in South Africa, LGBTQIA+ individuals continue to experience systemic discrimination and a heteronormative healthcare framework that challenge in accessing and utilizing the SRH services [19]. These challenges include stigma and discrimination [20-22], prejudice, unsupportive structural, and limited SRH services specific to their needs such as gender-affirming, adoption, and fertility services [23-26].

This does not only affect LGBTQIA+ individuals but also HCPs, as they have limited knowledge, skills, and expertise regarding SRH for LGBTQIA+ individuals [22-25]. Most HCPs in South Africa are not equipped with the type of SRH services related to LGBTQIA+ individuals due to their previous educational curriculum and lack of resources to provide relevant SRH services for LGBTQIA+ individuals [23,24,27-30]. The above challenges not only affect South Africa but also global and African areas [31-38].

By introducing easy, cost-effective, and user-friendly innovative solutions such as mHealth apps some of the above-mentioned challenges could be minimized and addressed. However, should the mentioned challenges not be addressed, LGBTQIA+ individuals could be at risk of negative health issues such as HIV or AIDS, STIs such as, mental health issues, and other health risks such as use of unregistered HCPs [39-44].

In South Africa, there is no specific developed mHealth app to be used by both the HCPs and LGBTQIA+ individuals for addressing SRH, thus improving the health and well-being of LGBTQIA+ individuals. Hence, our study developed an mHealth app prototype called *“Queery Wellness Hub”* to address the SRH of LGBTQIA+ individuals in Gauteng Province, South Africa. This article reports on how *“Queery Wellness Hub”* was developed using the Design Science Research (DSR) framework. The *“Queery Wellness Hub”* hopes to close gaps in the lack of knowledge, skills and expertise experienced by HCPs and address the SRH services for LGBTQIA+ individuals when accessing and utilizing these services.

## Methods

### Overview

**Figure 1.**
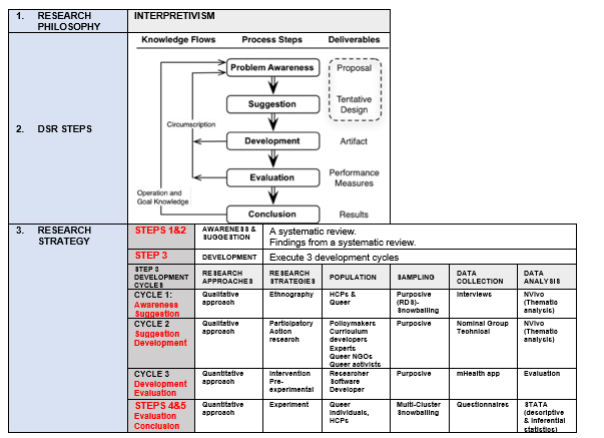
Design science research general steps [47].

This study reports the processes followed during the development of an mHealth app named *Queery Wellness Hub*. A detailed focus will mainly be on cycle three as it is the developed outcome and was not published anywhere, cycle one and two will be just a summary to show how these steps enhanced the final development. The Queer wellness hub was developed using Design Science Research (DSR) framework. The DSR framework was followed and applied as the main guide for the study. The principal investigator aimed at using the DSR framework developed by Kuechler et al [45]. This is an approach that follows sequential five steps namely, awareness, suggestion, development, evaluation, and conclusion. The DSR framework steps overlapped, and several iterations took place, especially during development [46]. As a result, the research questions and objectives were classified according to the DSR framework steps [47]. See Figure 1 for a summary of the DSR framework steps.

### Operationalization of Design Science Research (DSR) Framework

**Figure 2.**
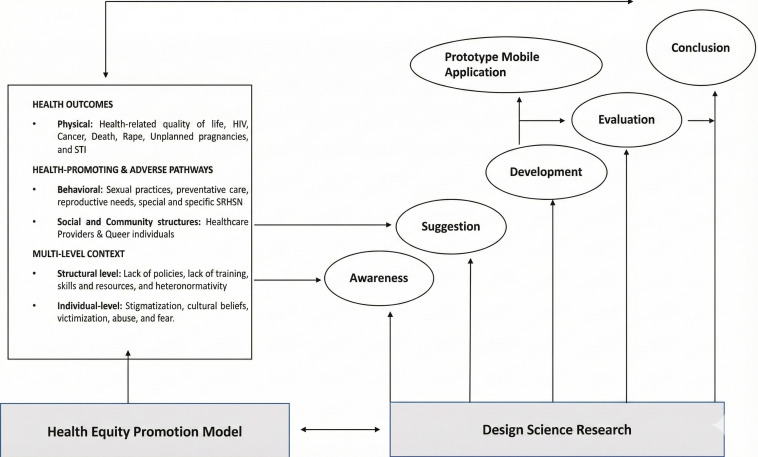
A proposed conceptual framework for developing a mHealth app for addressing SRH of LGBTQIA+ individuals adapted from HEPM & DSR.

This phase of the study used step three (developmental) of the DSR framework according to Vaishnavi and Kuechler [47]. This step comprised of three cycles. For clarity, while all cycles informed the development, this manuscript emphasizes the findings of cycle three, with cycles one and two described to show how they contributed to the ultimate development (Figure 2).

Cycle one covered step one (awareness), and two (suggestion) of the DSR framework. The awareness step covered the first three objectives, and the suggestion step covered last two objectives as described sub-section five. An ethnography qualitative approach was used during this cycle because it helped the principal investigator to deeply understand the specific sexual-reproductive health care needs of LGBTQIA+ individuals [27], find out the challenges faced by HCPs and LGBTQIA+ individuals [24], identify solutions to address the identified challenges [48], and explore their thoughts on using web-based tools and mHealth apps to address these needs [49]. 33 HCPs were selected across seven district hospitals in Gauteng province using purposive sampling as they were the relevant participants to provide rich information about provision of SHR, whereas 22 LGBTQIA+ individuals were selected from the one nongovernmental organization (NGO) which had two clinics specializing in health using respondent-driven sampling (RDS). The principal investigator started with two LGBTQIA+ members attending the clinic, then asked them to refer other LGBTQIA+ individuals until data was saturated (See the demographic data of the LGBTQIA+ individuals in MultimediaAppendices 1  2). For both participants, data was collected in private rooms using unstructured interview guide and all the interviews’ sessions were recorded using digital recorder. Interviews for HCPs were mainly in English as most of them were familiar with the language and for LGBTQIA+ individuals, they included a mixture of English and Isizulu which was a commonly used language and was later translated. Thematic analysis was used to analyze cycle one data using NVivo software 14.

Cycle two focused on the DSR framework step two (suggestion) which included the findings and recommendations from the experts and three (development) which involved a group of experts voting and ranking for the important content that needed to be included in the mHealth app development through the use of NGT. This cycle used a qualitative approach, specifically participatory action research (PAR) which helped with identifying the important SRH content to be included in a mHealth app thus assisted with the creation of a mHealth app to address SRH for LGBTQIA+ individuals. This qualitative approach allowed for a more in-depth understanding of the specific SRH topics that were to be considered when designing a mHealth app, assuring its relevance and efficacy in meeting the needs of LGBTQIA+ individuals seeking SRH [50]. This approach was executed in the form of NGT, a face-to-face workshop was conducted where all four NGT steps by were applied [51]. The NGT included participants such as the principal investigator, queer activists, sexual and reproductive health experts, private practicing health care providers, innovators, and private health care stakeholdersMultimedia Appendix 3. These participants were recruited via emails, from different social media platforms, and scholarly platforms based on their research interest and focus. The NGT had one moderator, two research assistants, and one principal investigator. The workshop lasted approximately 2 hours 46 minutes and 55 seconds. NGT was well-suited to this goal since it actively engaged the group of experts as co-principal investigator, allowed them to contribute their thoughts, experiences, and needs during the identification of the specific SRH topics for LGBTQIA+ individuals that needed to be included in a mHealth app(Multimedia Appendix 4).

The last cycle was cycle three. The cycle involved the prototype development guided by findings from both cycle one and two. This cycle encompassed the principal investigator and software engineers. This cycle applied a quantitative approach because the principal investigator was interested in the development of a prototype mHealth app. An interventional pre-experimental approach was used, which helped with ensuring that there are accuracy and inclusiveness of the prototype’s contents. After synthesizing and merging findings from cycles one and two, cycle three commenced, whereby it focused on the mHealth app prototype development. The principal investigator searched for software developers, managed to find one, and then nominated them as the developer based on their work experience. Data collection involved documenting and confirming the synthesized findings from the cycle one and two, with the principal investigator assessing clinical content and the software developer assessing technical functionality and interface. Both the developer and the principal investigator had regular face-to-face meetings which facilitated measurement and verification of the prototype’s features, effectively applying quantitative evaluation to the development process. The following steps were followed and repeated when developing the prototype: principal investigator compiled findings from cycles 1 and 2 and communicated them with the software developer; the prototype plan was conceptualized by both the principal investigator and the software developer, the software developer provided feedback on the design of the essential software components and the integration of functionalities and their potential impacts in addressing SRH for LGBTQIA+ individuals.

### Conceptual Framework of the Study

This study has adapted its conceptual framework from two theoretical models. Firstly, the Health Equity Promotion Model (HEPM) ensures the elimination of disparities in healthcare. For example, this model was used by Fredriksen-Goldsen [52] in their study to reconceptualize the health disparities of some LGBTQIA+ individuals. As a result, this model formed the basis for promoting SRH, thus answering the research questions of the specific SRH needs and the challenges experienced by both HCPs and LGBTQIA+ individuals.

Moreover, the proposed conceptual framework for developing a mHealth app for addressing the SRH of LGBTQIA+ individuals will use the DSR steps as the second framework and main guide for the study. This was due to the steps’ widespread use as a problem-solving technique for creating innovative artifacts such as healthcare mHealth apps [53]. For example, the DSR framework steps were used by a few studies in addressing health care issues such as mental health, and noncommunicable diseases [54,55].

In addition, this method assisted in answering and addressing almost all the research questions, but the study applied only three stipulated steps awareness, suggestion, and development. These steps are interrelated and overlap with each other. For example, *awareness and suggestion* were included in cycle one, *suggestion, and development* included in cycle two, and *development and evaluation* were covered after cycle three. Last, *evaluation and conclusion* are currently running as a post-doctoral project to test the prototype in step four, and five, and will take it back to *awareness****.***

### Ethical Considerations

The study received ethical approval from the Sefako Makgatho Health Sciences University Research Ethics Committee (SMUREC) (Reference Number: SMUREC/H/291/2023:PG) after being presented to and approved by the Department of Public Health Research Committee (DPHRC) and the School of Healthcare Sciences Research Committee (SHRC). Following ethical approval, the principal investigator obtained permission to conduct the study at selected Gauteng District hospitals through the National Health Research Database (NHRD), and separately requested permission from the POP INN clinic’s institutional management using the same clearance.

Written informed consent was obtained from all participants, who were informed about the study’s aims, objectives, and their rights, including the voluntary nature of participation and the freedom to withdraw at any point without negative consequences. Participants’ identities were protected through the use of pseudonyms and numerical codes (eg, P1 for Participant 1, E1 for Expert 1), and no names or surnames were recorded in any data tools, including interview guides and recordings.

To ensure privacy, confidentiality, and compliance with the Protection of Personal Information Act (POPIA), only minimal personal information was collected. All data were securely stored under lock and will be retained for five years, with controlled access. Participants’ personal information was not shared with third parties, and study findings were only shared with supervisors, transcribers, and through accredited journals or conferences. Experts who participated in workshops were also informed of their rights and were reimbursed R1000 (US $67) for transportation and fuel costs.

## Results

### Summary of Cycle Three Findings

The following information is about the descriptions of the developed mHealth app (Prototype) and its potential of addressing identified SRH for LGBTQIA+ individuals.

### About *Queery Wellness Hub*

*Queery Wellness Hub* is a health care hub that aims at addressing the SRH needs for the LGBTQIA+ individuals in Gauteng Province, South Africa. It is intended to be used by HCPs during health care provision, clients’ consultations, referrals, and as an information hub. Additionally, it is intended to be used by LGBTQIA+ individuals when accessing their SRH. An overall aim of the *Queery Wellness Hub* app is to address the issues of inclusivity and accessibility to health care for LGBTQIA+ individuals.

### How Does *Queery Wellness Hub* Work?

*Queery Wellness Hub* is designed to provide a centralized, accessible, and reliable source of health care information for queer individuals. It aims to create a supportive and informative space where users can access expert-verified content, connect with like-minded individuals, and engage with topics relevant to their health and well-being.

### Structure of the App (*Queery Wellness Hub*)

- Communities: “Communities” act as the information seeking part of the app
- Health care: This is where users are able to access a network of healthcare practitioners who provides all queer-related sexual and reproductive healthcare services and needs.
- Messaging: Here, users are able to communicate with practitioners and have in-app consultations
- Profile Management: Users are able to manage their profile and app preferences

### How Was *Queery Wellness Hub* Developed?

The app was developed from scratch through an empirical study using the *Design Science Research (DSR) framework* steps by Vaishnavi & Kuechler [47]. The main developer of the app is Dr Raikane James Seretlo. The principal investigator applied various series of cycles to develop this app, including a systematic review, qualitative approaches with HCPs and LGBTQIA+ individuals were included, a group of experts, and a software developer. The findings from the empirical cycles recommended the SRH topics that should be included in the app.

### What Are the Benefits of *Queery Wellness Hub*?

*Queery Wellness Hub* is intended to be used by different HCPs as a consultative resource whenever they are in contact with LGBTQIA+ individuals. It may support sexual and reproductive health (SRH) of LGBTQIA+ individuals and aims to enhance equitable access to healthcare. The app could contribute to supporting SRH knowledge and service uptake among LGBTQIA+ individuals. The app may also support HCP workflow and clinical decision-making by providing a consultative resource and facilitating access to information tailored to LGBTQIA+ individuals SRH.

### What’s Next for *Queery Wellness Hub*?

The post-doctoral project commenced to test *Queery Wellness Hub* for addressing SRH by both HCPs & LGBTQIA+ individuals. Evaluation of the effectiveness and functionality of *Queery Wellness Hub* in addressing SRH for LGBTQIA+ individuals. Further research is needed to study the impact of Queery: Wellness Hub on health behaviors and clinical outcomes.

### Visuals of the Developed mHealth App (Prototype)

This section provides visuals of the developed mHealth app (prototype), illustrating the main outcome of the study. Not all pictures of a developed mHealth app (prototype) were included in this section, this is a summary of and visual presentation of how a developed mHealth app (prototype).

#### Onboarding and Access

**Figure 3.**
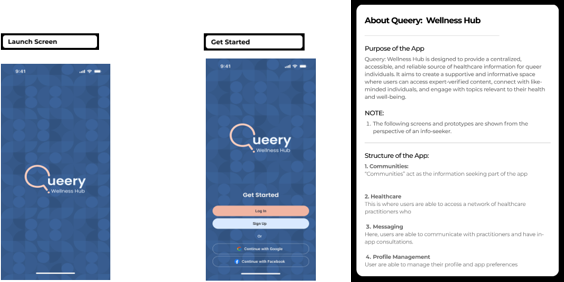
This screen allows users to enter their personal log-in details to access the app securely. In addition, the “About” section provides a brief background regarding the *Queery Wellness Hub*, giving users an overview of its purpose and features before they begin using it.

The app welcomes users with a simple and secure login interface (Figure 3), providing an easy point of access while ensuring confidentiality. This login screen serves as the digital gateway to a personalized LGBTQIA+ health journey.

#### Holistic SRH Information Hub

**Figure 4.**
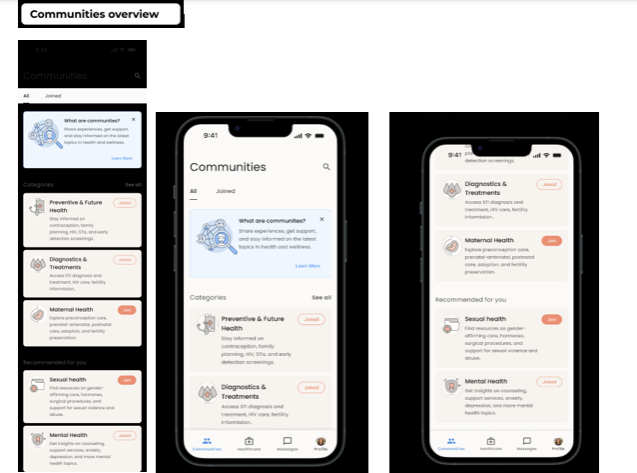
The information hub icon provides access to all sexual and reproductive health (SRH) information tailored for LGBTQIA+ individuals. It also includes different community spaces where healthcare professionals (HCPs) and LGBTQIA+ users can engage with other members of the app to discuss various health categories. Additionally, users can search for specific information related to any health-related matters through this hub.

Once logged in, users are directed to a central SRH information hub (Figure 4). This section allows individuals to access curated and diverse resources including preventive care, diagnostic services, maternal and sexual health education, and mental health support. Communities within the app are thematically organized to cater to various SRH interests and needs, enhancing relevance and engagement. Additionally, users can join different communities, providing them with various SRH data such as preventive & future health, diagnostic treatments, maternal health, sexual health, and mental health.

#### Community Interaction and Peer Support

**Figure 5.**
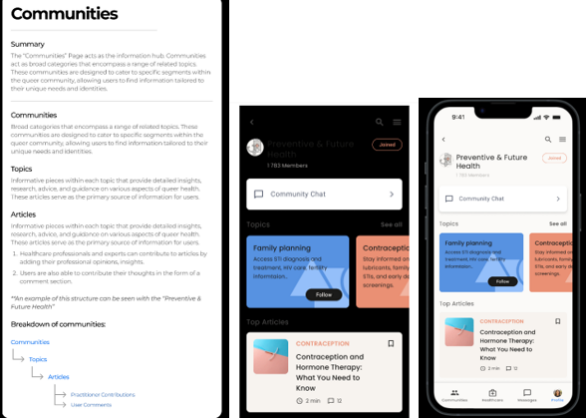
This section includes several topics and articles, such as those specific to LGBTQIA+ individuals and holistic SRH, including family planning, preventative health, and future health. In addition, a chat column is provided to enable users to communicate and interact with each other within the community.

The app promotes open and stigma-free conversations through peer-led communities (Figure 5). LGBTQIA+ users can engage in discussions on family planning, contraception, STI/HIV prevention, and other relevant topics. This platform also highlights top SRH-related articles, offering evidence-based knowledge to empower users in managing their health. Furthermore, LGBTQIA+ individuals will be able to join different communities and discuss various topics freely, such as family planning, contraception, and HIV and STI prevention (Figure 5). Additionally, they will be able to access top articles related to SRH and read more about them.

#### Article and Comment Section

LGBTQIA+ individuals will be able to chat among themselves and ask questions to the HCPs about all the matters relating to their SRH. This is in the form of support groups. Additionally, LGBTQIA+ individuals can access uploaded articles regarding their SRH and learn more about any of their sexual and reproductive health services and needs. This icon can also be used by the HCPs in terms of understanding the LGBTQIA+ individuals health requests and concerns.

#### Direct Chat With Health Professionals

**Figure 6.**
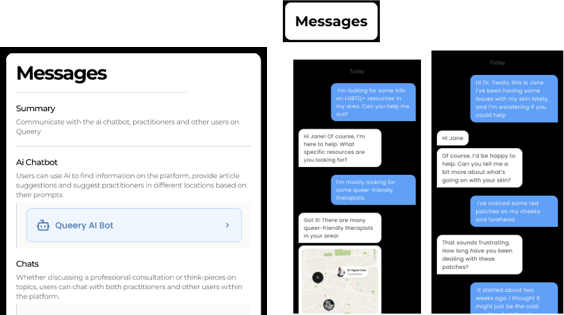
The Message icon allows LGBTQIA+ users to communicate directly with their healthcare providers (HCPs) regarding any health issues. HCPs can also use this feature to send reminders, share updates, and provide consultation details to LGBTQIA+ individuals. The AI Bot icon provides users with instant support through an automated virtual assistant, offering answers to common health-related questions, guidance on navigating the app, and suggestions for appropriate resources when needed.

Beyond peer interaction, the prototype includes a direct messaging feature (Figure 6) where users can confidentially engage with health care professionals (HCPs). This channel fosters real-time support, allows follow-up queries, and encourages ongoing health conversations tailored to queer identities. This section is about *Queery Wellness Hub*’s messaging and AI Bot, where LGBTQIA+ individual scan ask SRH questions and receive reminders for their consultations with HCPs. Messages can include AI bot, HCPs, and peers whenever seeking SRH advice.

#### Profile and Emergency Support

**Figure 7.**
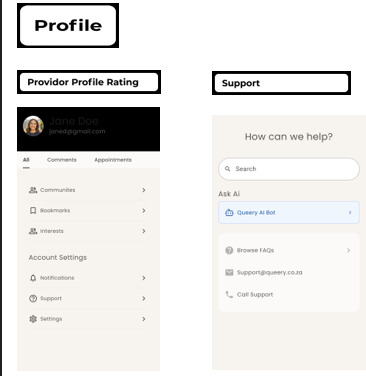
The Profile icon allows users to manage their personal information, preferences, and health-related details securely within the app. The Emergency Support icon provides immediate access to urgent resources and contacts, ensuring that LGBTQIA+ individuals can quickly reach help or support services during critical situations.

User security is prioritized through the profile section (Figure 7), which includes privacy settings and emergency contact options. It also provides shortcuts to book consultations or get immediate assistance bridging the gap between digital engagement and real-world support. In addition, will provide LGBTQIA+ individuals with their security details, options to check where they can call for support and details. Further, this icon will serve as a reminder and provide them with an option to contact HCPs for consultations. There is an AI chatbot and consultation reminders under this icon. This notable feature of *Queery Wellness Hub* is the AI-powered chatbot (Figure 6). This tool not only answers SRH questions instantly but also sends reminders for upcoming consultations, ensuring continuity of care. The chatbot interacts fluidly with users, HCPs, and peer groups, serving as a smart companion in their health journey.

#### Virtual Consultations

Within the profile, recognizing the importance of personal interaction the video call feature facilitates virtual consultations and group support meetings. This innovation brings healthcare access closer, especially for those in remote or underserved areas. This icon is also available for peers and consultations which can be utilized during consultation and support groups meetings.

#### Finding LGBTQIA+ Friendly HCPS

Finally, the “Find Healthcare Provider” function helps users locate affirming SRH service providers. LGBTQIA+ individuals can search for HCPs who are rendering specific SRH by clicking on the find healthcare provider. This icon will provide LGBTQIA+ individuals with the location and distance of the HCPs; contact details and the services they provide. LGBTQIA+ individuals will be able to book their appointment online. It shows a directory with distance, services, and contact details, and even allows appointment bookings, making healthcare access efficient and queer-friendly.

## Discussion

### Principal Findings

The findings of this study showed that Design Science Research (DSR) framework enabled the development of an mHealth app called *Queery Wellness Hub* through its interrelated steps. These steps include awareness, suggestion, development, and evaluation through three cycles. The study indicates that mHealth apps are ideally developed with a co-creation approach, involving users, subject experts, and software developers, to enhance relevance and usability. This finding is supported by the sequential mixed-methods approach employed in the study, including ethnography, PAR, and prototype development with software developers. Below will discuss the developed mHealth in comparison to the prior scholarly work.

### Comparison With Prior Work

Our study showed that *Queery Wellness Hub* mHealth app includes have an onboarding and access icon which welcomes all the users with a simple and secure login interface. Furthermore, our study emphasized this login screen to serve as a digital gateway to a personalized LGBTQIA+ health journey [56]. study emphasizes that app trustworthiness essential for initial user uptake relies on clear onboarding processes, transparency in data handling, and strong privacy features. Similarly, participants in the study [57] expressed anxiety about their privacy when using mHealth apps. They also revealed their preferences for various security aspects in mHealth apps, including regular password updates, remote erase, user consent, and access controls [57]. All these studies including ours suggest that security is an important aspect of mHealth app and this may influence on the effective use of mHealth apps. Therefore, principal investigator s, innovators, and developers should ensure that all mHealth app consist of the secure login interface to promote privacy.

This study revealed that *Queery Wellness Hub* mHealth app can offer and serve as holistic SRH information hubs, where LGBTQIA+ individuals can access arranged and varied services such as preventive care, diagnostic services, maternal and sexual health education, and mental health support. Our study is in line different studies conducted around SRH that showed an importance of mHealth app in offering and enhancing SRH knowledge, attitudes, and service uptake [58]; provide sexuality, HIV/STI education plus navigation to services [59]. Overall, these indicated how important mHealth app could assist with sharing different health information and render services such as preventive care, diagnostic services, maternal and sexual health education, and mental health support thus increasing knowledge and changing attitude towards SRH update.

On top of that, our study illustrated that *Queery Wellness Hub* mHealth app can have community interaction and peer support icons where LGBTQIA+ users can join different community and discuss the different topics freely like family planning, contraception, STI/HIV prevention, and other relevant topics. Our findings are consistent with a study conducted [60], which found that peer-led group facilitation enabled the provision of sensitive psychological support, allowing young people to express themselves freely, acquire a feeling of self-worth, and engage more. This aligns with previous findings, our study corresponds with [61] study, which demonstrated that the smartphone app includes a secure, anonymous community messaging board, allowing users to express stigma, receive peer reassurance, and support retention in care. Furthermore, a study by [62] found a great need for community-sharing venues in which users may publish, discuss, and exchange experiences, tales, and Q&A, providing a safe, culturally resonant atmosphere. Communication is one of the digital elements that could be improved [63]. Connection to other individuals with similar experiences via an online forum, community, or instant messages in the mobile tool, or by connecting with other kinds of social media [63]. Altogether, these studies show that a comment section which allows LGBTQIA+ individuals to engage and share their experiences about various health topics in an anonymous way could improve their health and wellbeing.

Article and comment section icons was one of the findings our study highlighted as important in the *Queery Wellness Hub* mHealth app inclusion. This icon will enable HCPs and LGBTQIA+ individuals to access any articles that is peer-reviewed and has relevant information regarding SRH services and needs. As well as having a comment section for LGBTQIA+ individuals to be able to chat for among themselves and ask questions to the HCPs about all the matters relating to their SRH. Our findings match with a [63] study, which stressed the features to better mHealth app interventions, highlighting communication as an essential component for giving access to a health care practitioner via email, text message, or live chat. Furthermore [64], emphasizes the need of two-way communication by providing forums or chat rooms where users can connect with one another and with providers. They also highlight the significance of reputable, evidence-based information. Furthermore [65], showed that the community message board enabling users to share experiences, questions, and support.

Direct chat with HCPs and use of AI bot were also found by our study during the development of *Queery Wellness Hub* mHealth app. These icons were developed to allow LGBTQIA+ individuals to communicate with HCPs real-time support, allows follow-up queries, and encourages ongoing health conversations tailored to LGBTQIA+ identities. In addition, these icons found to serve as a reminder for LGBTQIA+ individuals’ consultations with HCPs. Our study matches up with a study [66] that showed that direct messaging/chat with HCPs promotes patient engagement, provides for timely follow-up, and facilitates individualized health talks. Furthermore , presented apps with real-time chat and appointment reminders adapted to the requirements of sexual and gender minorities, demonstrating greater linkage to care and retention through reminders and direct provider connection [67].

Our study showed an importance of privacy by developing profile of each LGBTQIA+ individual’ user including section where they can receive emergency support when utilizing the *Queery Wellness Hub* app. This provide the LGBTQIA+ users with shortcuts to book consultations or get immediate assistance bridging the gap between digital engagement and real-world support. Our findings correspond with [68], who used the Vula app and demonstrated that it enabled emergency clinicians to consult burn specialists via mobile phone, providing photos and patient information. Some research found that booking or seeking specialist consultations via an app can enhance results for patients [68,69]. Consistently, our findings is consistent with [70], which emphasizes that real-time booking and reminders improve patient-provider collaboration and access, particularly in resource-limited situations.

Additionally, our study demonstrated that once the LGBTQIA+ users gets inside their profile they can easily arrange and have virtual consultations with HCPs via video, thus bringing healthcare access closer, especially for those LGBTQIA+ users in remote or underserved areas. Our study displayed that this icon can also be used to by peers during consultation and support groups meetings. Our findings are consistent with [60] peer-led mobile support system (chat-based), which successfully boosted mental health and engagement, laying the framework for peer-enabled video sessions. Additionally [71], demonstrated that video-based teletherapy met both individual and peer-group health needs among LGBTQ+ young people.

Lastly, our study showed that the *Queery Wellness Hub* app could offer LGBTQIA+ users assistance in finding LGBTQIA+ friendly HCPs. This means that locating affirming SRH service HCPs through clicking find healthcare provider button. In addition, once they find the HCP, LGBTQIA+ users will be able to see location and distance of the HCPs; contact details and the services they provide, and able to book their appointment online. Our study is in accordance with a randomized controlled trial evaluated an app that tracked users via GPS and featured a clinic finder tool that mapped nearby clinics to assist pregnant and postpartum women living with HIV in accessing care [72,73]. Some studies showed that mHealth app helped with online appointment systems that allow users to search for HCPs by area, check availability, and schedule appointments immediately [72,73]. A recent evaluation of Cameroon-based apps included Healthlate, which allows appointment booking, in-app conversation with doctors, and has integrated location-based provider listings [74]. All statements regarding the app’s features, such as secure login, community interaction, article access, AI bot, direct HCP communication, privacy protections, virtual consultations, and HCP finder functionalities, are grounded in the empirical data collected through cycles one and two (ethnographic interviews and NGT workshops) and operationalized in the prototype development of cycle three.

### Limitations

Despite the benefits of RDS, the sample was drawn from a single NGO in Gauteng Province, South Africa which may not reflect the full diversity of LGBTQIA+ individuals experiences in other regions. Several sub-groups such as lesbians, bisexuals, intersex persons, men who have sex with men (MSM), and those identifying as pansexual or falling under the “plus” spectrum were underrepresented due to the hidden nature of the participants, difficult to reach in public health facilities, and some logistical challenges. Although interviews conducted in isiZulu and Sesotho were translated into English, some nuances may have been lost, potentially affecting the depth of meaning in the responses.

There is also a risk of social desirability bias, both participants both LGBTQIA+ individuals and HCPs may have offered responses they deemed acceptable rather than entirely truthful. While the expert workshop brought valuable input, the withdrawal of 12 invited participants due to work and family obligations reduced the diversity of expert opinions, although those who participated still represented broad expertise. Lastly, while mHealth apps present a promising solution for improving SRH access and support, their implementation and impact were not tested in this study and should be explored further in future research.

### Recommendations

The developed mHealth app prototype should undergo rigorous testing and evaluation to assess usability, effectiveness, and user acceptance. Researchers can apply frameworks such as the Unified Theory of Acceptance and Use of Technology (UTAUT) to guide these evaluations. In additional, digital experts, engineers, LGBTQIA+ health specialists, and healthcare practitioners, and the National Department of Health should be engaged to refine and scale the app, ensuring it meets the diverse needs of users. Further, adequate funding from multiple sources is recommended to support the expansion, functionality, and sustainability of the mHealth app.

### Future Directions

A shift from non-functional policies, strategies, and frameworks should be highlighted and practiced by different scholar. As a result, future research should be focused on studies that will enhance and introduce unique innovative solutions and technologies to improve the health of the LGBTQIA+ individuals, hence enhancing equality within the country and African continent.

### Conclusions

Our study achieved its main purpose, that was, to develop an mHealth app to address SRH for LGBTQIA+ individuals in Gauteng Province, South Africa. These findings suggest that technology-based solutions can help mitigate some social, physical, psychological, and sexual health challenges. The findings from our study show an importance of how innovative solutions could be implemented for strengthening the healthcare sector and closing some of the disparities experienced by LGBTQIA+ individuals. Therefore, all details about the app’s usefulness and relevance for LGBTQIA+ individuals can be reasonably drawn from the collected data and observed prototype outcomes.

## Supplementary material

10.2196/79593 Multimedia Appendix 1 Summary of cycle one demographic data and findings. This table summarizes the objectives, participant demographics, and key thematic findings from cycle one research data related to LGBTQIA+ sexual-reproductive healthcare. Combined overall summary of the cycle one details. All these findings were published in 2024, and references are given under each objective to avoid duplication.

10.2196/79593 Multimedia Appendix 2 Summary of demographic data for both HCPs and LGBTQIA+ individuals in cycle 1. This table presents the demographic data collected from both groups of participants, including all the basic demographic information.

10.2196/79593 Multimedia Appendix 3 Summary of cycle two demographic data and findings. Demographic characteristics of all consulted experts for mHealth App development. Experts identified the following pillars as crucial to the design and implementation of the mHealth app. Each pillar was ranked based on relevance, practicality, and user-centeredness. Similarly to cycle one, this cycle details are just a summary to orientate the reader of what led to cycle three development.

10.2196/79593 Multimedia Appendix 4 Thematic Priorities for mHealth App Content. This includes all important contents that experts voted and ranked to be included in the mHealth prototype.

## Abbreviations

AI: Artificial Intelligence
CEOs: Chief Executive Officers
DHET: Higher Education and Training
DPHRC: Department of Public Health Research Committee
DSR: Design Science Research
HbA1c: Glycosylated hemoglobin
HCPs: Healthcare providers
HEPM: Health Equity Promotion Model
HICs: High-income countries
HIV: Human immunodeficiency virus
LGBTQIA+: Lesbian, gay, bisexual, transgender, queer, intersex, asexual, and plus
LICs: Low-income countries
mHealth app: Mobile health application
Mobile Health: mHealth
MSM: Men who have sex with men
nGAP: New Generation of Academics Programme
NGO: Non-governmental organization
NGT: Nominal Group Technique
NHI: National Health Insurance
NHRD: National Health Research Database
NRF: National Research Foundation
PAR: Participatory action research
POPIA: Protection of Personal Information Act
PrEP: Pre-Exposure Prophylaxis
RDS: Respondent-driven sampling
SHRC: School of Healthcare Sciences Research Committee
SMUREC: Sefako Makgatho Health Sciences University Research Ethics Committee
SRH: Sexual and reproductive health
SSAUF: Staffing South Africa's Universities Framework
STI: Sexually transmitted infection
UTAUT: Unified Theory of Acceptance and Use of Technology
