# Supplementary material for: Development of an mHealth App Prototype for LGBTQIA+ Individuals’ Sexual and Reproductive Health in Gauteng Province, South Africa: Design Science Research Study
Source: JMIR Form Res. 2025 Dec 23;9:e79593. doi: 10.2196/79593 (PMC12724484; doi:10.2196/79593)
Supplement: Multimedia Appendix 1 [file formative-v9-e79593-s001.pdf]

Objective	Participant Demographics	Summarized Thematic Findings
Understand specific sexual-reproductive healthcare needs of LGBTQIA+ individuals by HCPs [27].	33 HCPs (mostly female, ages 23–57.<br><br>Social workers, nurses, psychologists, doctors).<br><br>Work experience: 2–36 years.	Need for inclusive, queer-friendly healthcare environments.<br><br>Psychological and counselling support essential.<br><br>Access to sexual/reproductive education.<br><br>Recognition of queer-specific reproductive health services
Identify challenges faced by both HCPs and LGBTQIA+ individuals [24].	33 HCPs: 85% female, all straight.<br><br>22 LGBTQIA+ individuals: 86% male, diverse sexual orientations & diverse gender expressions.<br><br>Majority single (LGBTQIA+ individuals), mixed marital status (HCPs).	LGBTQIA+ individuals fear stigma and negative attitudes from HCPs.<br><br>HCPs feel confused/unprepared about gender identity.<br><br>Mutual barriers between LGBTQIA+ individuals and providers.<br><br>Healthcare disparities and familial challenges
Explore queer individuals' perceptions and solutions for improving SRH [48].	22 LGBTQIA+ individuals.<br><br>Mean age ~26, mostly male with diverse queer identities.<br><br>All single, education secondary/tertiary.	Create healthcare equity and LGBTQIA+ individuals-specific clinics.<br><br>Empower and train HCPs in LGBTQIA+ individuals health care.<br><br>Raise public awareness about queer issues.<br><br>Engage political leaders, community, and LGBTQIA+ individuals.
Explore perspectives of HCPs and LGBTQIA+ individuals on web-based tools and mHealth apps [49].	33 HCPs: Mean age 36.7, mostly female.<br><br>22 Queer individuals: Mean age 25.9, mostly male with queer identities.<br><br>Varied education levels in both groups.	Digital tools can facilitate consultations and treatment access.<br><br>Positive attitudes toward mobile health apps and technology.<br><br>Technology is seen as empowering and time-saving.<br><br>A Need to enhance digital literacy and knowledge sharing.

- ***Straight in this study: refers to heterosexuals***
