# Supplementary material for: Development of an mHealth App Prototype for LGBTQIA+ Individuals’ Sexual and Reproductive Health in Gauteng Province, South Africa: Design Science Research Study
Source: JMIR Form Res. 2025 Dec 23;9:e79593. doi: 10.2196/79593 (PMC12724484; doi:10.2196/79593)
Supplement: Multimedia Appendix 2 [file formative-v9-e79593-s002.pdf]

| **Characteristics** | **HCPs** ***n (=33)*** | **Queer people** ***n (=22)*** |
|---|---|---|
| **Age, Mean (SD)** | **4.96 (1.84)** | **2.86 (.94)** |
| **Birth gender *n (%)*** | | |
| *Male* | **5 (15.15)** | **19 (86.36)** |
| *Female* | **28 (84.85)** | **3 (13.64)** |
| **Sexual orientation *n (%)*** | | |
| *Straight* | **33 (100)** | **0** |
| *Lesbian* | **0** | **3 (13.64)** |
| *Gay* | **0** | **8 (36.36)** |
| *Bisexual* | **0** | **2 (9.09)** |
| *Transgender woman* | **0** | **7 (31.82)** |
| *MSM* | **0** | **2 (9.09)** |
| **Marital status *n (%)*** | | |
| *Single* | **22 (66.67)** | **22 (100)** |
| *Married* | **11 (33.33)** | **0** |
| **Education level *n (%)*** | | |
| *No formal education* | **0** | **0** |
| *Primary* | **0** | **0** |
| *Secondary* | **0** | **10 (45.45)** |
| *Tertiary* | **33 (100)** | **12 (54.55)** |

- ***Straight in this study: refers to heterosexuals***
