# Supplementary material for: Development of an mHealth App Prototype for LGBTQIA+ Individuals’ Sexual and Reproductive Health in Gauteng Province, South Africa: Design Science Research Study
Source: JMIR Form Res. 2025 Dec 23;9:e79593. doi: 10.2196/79593 (PMC12724484; doi:10.2196/79593)
Supplement: Multimedia Appendix 3 [file formative-v9-e79593-s003.pdf]

Expert No.	Age	Sex / Pronouns	Race	Focus Area
1	31	Male / He/Him	African	Sexuality, LGBTQIA+, masculinity, and gender
2	28	Female / She/Her	African	Innovative marketing
3	55	Female / She/Her	African	STIs and key populations
4	39	Male / He/Him	African	HIV, TB and key populations
5	35	Male / He/Him	African	Health communication and LGBTQIA+
6	29	Male / He/Him	African	Key population research
7	29	Female / She/Her	African	Primary healthcare
8	24	Male / He/Him	African	Data analytics
9	34	Female / She/Her	African	Psychosocial sciences
10	27	Female / She/Her	Coloured	Communications
11	55	Male / He/Him	White	SRH and LGBTQIA+
12	48	Male / He/Him	African	OBGYN and principal investigator
13	57	Female / She/Her	African	Sexual reproductive health

"*In the South African context, 'Coloured' refers to a multiracial ethnic group with diverse ancestral heritage and is recognized as a distinct demographic category in official statistics.*"
