# Supplementary material for: Development of an mHealth App Prototype for LGBTQIA+ Individuals’ Sexual and Reproductive Health in Gauteng Province, South Africa: Design Science Research Study
Source: JMIR Form Res. 2025 Dec 23;9:e79593. doi: 10.2196/79593 (PMC12724484; doi:10.2196/79593)
Supplement: Multimedia Appendix 4 [file formative-v9-e79593-s004.pdf]

Pillar	Summary of Key Contents / Features
**User Demographics**	Profile creation includes pronouns, gender identity, and age; facility locator feature.
**Features**	App tools like online consultation, AI chatbot, videos, share button, support contact.
**Accessibility and Inclusivity**	Ensure usability for people with disabilities and varied backgrounds.
**Connectivity**	App availability offline and for users without smartphones via USSID access.
**Privacy and Security**	Secure login, user agreements, regular password changes, and data protection.
**Functionalities**	Push notifications including SMS reminders.
**Service Integration & Partnerships**	Links with HCPs, labs, public and private health services.
**User Engagement**	Referral options, FAQs, and peer support groups.
**Information Hub**	Comprehensive sexual and lifestyle health education, including gender-affirming services.
